# Fractal Dimension in Quantifying Experimental-Pulmonary-Hypertension-Induced Cardiac Dysfunction in Rats

**DOI:** 10.5935/abc.20160083

**Published:** 2016-07

**Authors:** Francis Lopes Pacagnelli, Ana Karênina Dias de Almeida Sabela, Thaoan Bruno Mariano, Guilherme Akio Tamura Ozaki, Robson Chacon Castoldi, Edna Maria do Carmo, Robson Francisco Carvalho, Loreta Casquel Tomasi, Katashi Okoshi, Luiz Carlos Marques Vanderlei

**Affiliations:** 1 Pós Graduação em Ciência Animal, Curso de Fisioterapia, UNOESTE, Presidente Prudente, São Paulo - Brazil; 2 Programa de Pós Doutorado, FCT/UNESP, Presidente Prudente, São Paulo - Brazil; 3 Departamento de Fisioterapia, FCT/UNESP, Presidente Prudente, São Paulo - Brazil; 4 Departamento de Educação Física UNOESTE, Presidente Prudente, São Paulo - Brazil; 5 Departamento de Morfologia, UNESP, Botucatu, São Paulo - Brazil; 6 Faculdade de Medicina, UNESP, Botucatu, São Paulo - Brazil

**Keywords:** Heart Failure/mortality, Hypertension, Pulmonary, Cardiomegaly, Rats, Echocardiography/methods, Monocrotaline

## Abstract

**Background:**

Right-sided heart failure has high morbidity and mortality, and may be caused by pulmonary arterial hypertension. Fractal dimension is a differentiated and innovative method used in histological evaluations that allows the characterization of irregular and complex structures and the quantification of structural tissue changes.

**Objective:**

To assess the use of fractal dimension in cardiomyocytes of rats with monocrotaline-induced pulmonary arterial hypertension, in addition to providing histological and functional analysis.

**Methods:**

Male Wistar rats were divided into 2 groups: control (C; n = 8) and monocrotaline-induced pulmonary arterial hypertension (M; n = 8). Five weeks after pulmonary arterial hypertension induction with monocrotaline, echocardiography was performed and the animals were euthanized. The heart was dissected, the ventricles weighed to assess anatomical parameters, and histological slides were prepared and stained with hematoxylin/eosin for fractal dimension analysis, performed using box-counting method. Data normality was tested (Shapiro-Wilk test), and the groups were compared with non-paired Student t test or Mann Whitney test (p < 0.05).

**Results:**

Higher fractal dimension values were observed in group M as compared to group C (1.39 ± 0.05 vs. 1.37 ± 0.04; p < 0.05). Echocardiography showed lower pulmonary artery flow velocity, pulmonary acceleration time and ejection time values in group M, suggesting function worsening in those animals.

**Conclusion:**

The changes observed confirm pulmonary-arterial-hypertension-induced cardiac dysfunction, and point to fractal dimension as an effective method to evaluate cardiac morphological changes induced by ventricular dysfunction.

## Introduction

In experimental models, structural cardiac changes are usually identified by use of morphometric and/or histological analyses.^1-3^ Ventricular weight normalized to final body weight (FBW), obtained via morphometric analysis, has been used to characterize ventricular hypertrophy,^4-7^ while histological analysis has been used to characterize cardiac changes qualitatively, for example, inflammatory process, or quantitatively, by measuring cardiomyocyte area, blood vessels and interstitium.^1,6^

Another way to identify structural changes is fractal dimension, which allows the characterization of irregular structures in histological slides and the quantification of changes.^8-12^

To determine fractal dimension with histological analysis, box-counting is one of the most used techniques.^13^ It consists in sliding an r-sided square box over an image in an overlapping pattern, the square side r being progressively smaller; Nr is the number of r-sided squares necessary to overlap the image, at each side size chosen. Fractal dimension is the inclination of the regression line for the log-values of the box size (r) and the number of squares (Nr).^13^

Fractal dimension has been used as a diagnostic tool for retinopathies, histopathological studies of neoplasms, morphometry of liver cells, liver fibrosis and cardiac studies.^9,11,14^ In addition, it has been used to assess the left ventricle of patients submitted to cardiac transplantation, contributing to quantify myocardial cellular rejection.^13^ However, to our knowledge, there is no study assessing fractal dimension in pulmonary arterial hypertension (PAH), a disease that can cause structural right ventricular (RV) modifications, inducing ventricular functional changes that affect the patients' functional capacity and quality of life.^15,16^ Of the experimental models to induce PAH, the one with monocrotaline, described by Lalich and Merkow in 1961, stands out; it is used to cause ventricular hypertrophy, RV dysfunction and heart failure.^6,17-21^ Monocrotaline is a pyrrolizidine alkaloid found in plants of the *Crotalaria spectabilis* species, which causes pulmonary endothelial injury, an increase in vasoconstrictors and thickening of vascular wall (mainly smooth muscle cells), leading to an increase in pulmonary resistance and RV overload.^22,23^

Method studies that, alone or along with cardiac histological assessment, can contribute to increase the accuracy in diagnosing ventricular dysfunction caused by PAH are fundamental.^24^ Right ventricular assessment in PAH by using fractal dimension can be useful to evaluate the physiopathology, as well as the influence of therapeutic interventions in that condition.

This study was aimed at assessing the use of fractal dimension in the cardiomyocytes of rats with monocrotaline-induced PAH, associated with histological and functional analysis. The hypothesis is that PAH induces ventricular dysfunction, which can be identified by use of fractal dimension.

## Methods

### Animals

This study used 16 adult male Wistar rats, aged 4 months, weighing 358.5 g (±16.26 g), from the central vivarium of the Oeste Paulista University (UNOESTE), Presidente Prudente city, São Paulo. The animals were maintained in the Animal Experimentation Laboratory of the same institution, in plastic boxes measuring 41x34x16 cm (3 animals/box), at a temperature of 21°C to 23°C, and 50% to 60% relative humidity, with 12 hour light/dark cycles, light beginning at 7AM. The animals received water *ad libitum* and food preparation (Supralab, Alisul®, Brazil) proportionally to the amount consumed in the group treated with monocrotaline.

All experimental procedures used in this study abided by the principles of care for laboratory animals of the Brazilian College for Laboratory Animals (*Colégio Brasileiro de Experimentação Animal* - COBEA), in accordance with the Guide for the Care and Use of Laboratory Animals published by the National Research Council.^25^ This study was approved by the Ethics Committee of the UNOESTE (Protocol 1838).

### Experimental design

Initially the animals were randomly distributed into two groups with eight animals each: control (C) group and monocrotaline (M) group. Group M animals received one single intraperitoneal dose of monocrotaline (60 mg/kg - Sigma Chemical, St Louis, MO, USA), while group C animals received an intraperitoneal saline solution injection (0.9% NaCl).

After 5 weeks, the rats underwent echocardiographic assessment, which identified PAH and RV dysfunction in group M. After that, the animals were weighed and euthanized with an overdose of sodium pentobarbital (50 mg/kg). The heart was removed, dissected and weighed. Then, histological slides were prepared for histological and fractal dimension assessment.

### Induction of pulmonary hypertension

The protocol for PAH induction was performed in group M animals with injection of one single intraperitoneal dose of monocrotaline (Sigma Chemical, St Louis, MO, USA) at the proportion of 60 mg/kg in 1 mol/L in HClph buffer 7.0 with 1 mol/l of NaOH.^19^

After receiving monocrotaline, the animals were separated into individual cages to measure their daily consumption of food preparation. Group M animals were fed *ad libitum*; however, their food preparation intake decreased because of RV dysfunction. Therefore, group C animals received the mean amount of food preparation consumed by group M animals.

Group C animals underwent an intraperitoneal saline solution injection (0.9% NaCl), to ensure all study animals would undergo the same degree of stress.

### Echocardiographic functional assessment

The M mode echocardiography was performed with an echocardiographic device (Philips®, model HDI 5000, Netherlands) equipped with a 12 MHz electronic transducer,^26^ and the animals were anesthetized with intraperitoneal ketamine (60 mg/kg) and xylazine (1 mg/kg) hydrochloride.

Table 1 shows the parameters assessed: pulmonary acceleration time (PAT), maximum pulmonary artery velocity (PAVm) and ejection time (EJT).^27^

**Table 1:** Right ventricular echocardiographic parameters expressed as mean ± standard deviation, median, minimum and maximum values of the groups studied

Variables	Group C	Group M	p value
PAVm (cm/s)	88.50 ± 4.68 (87.50) [81.00– 97.00]	69.33 ± 18.17 (74.00) [36.00– 78.00]	0.0275
PAT (ms)	29.00 ± 3.16 (29.00) [26.00–33.00]	21.00 ± 3.80 (22.00) [15.00–26.00]	0.0005
EJT (ms)	86.33 ± 3.26 (87.00) [81.00–89.00]	75.44 ± 9.81 (78.00) [63.00 –89.00]	0.022

C: control; M: monocrotaline; PAVm: maximum pulmonary artery velocity; PAT: pulmonary acceleration time; EJT: right ventricular ejection time; cm/s: centimeters per second; ms: milliseconds. (PAVm: non-paired t test; PAT and EJT: Mann-Whitney test)

### Assessment of anatomic parameters

To assess the anatomic parameters, the heart was removed and dissected, and the atria (ATs) and right and left ventricles were separated and weighed. Humid right and left atrial weights and right and left ventricular weights (RAW, LAW, RVW and LVW, respectively) were normalized to the animal's FBW (RAW/FBW, LAW/FBW, RVW/FBW and LVW/FBW, respectively) and used as ventricular hypertrophy indices.^4^

### Histology and histomorphometric analysis

Samples of the right ventricle were fixed in a 10% buffered formalin solution for 48 hours. After fixation, the tissue was embedded in paraffin blocks, and later two 4-micrometer coronal histological sections were obtained for each animal. The histological sections were stained with Hematoxylin-Eosin (HE) solution and mounted on glass slides. The cross-sectional areas of cardiomyocytes were assessed by using a LEICA microscope (DM750 model, Germany) coupled to a video camera that sends digital images to a computer equipped with a program of image analysis (Image Pro-plus - Media Cybernetics, Silver Spring, Maryland, USA).^28,29^

All images were captured at 40x magnification. The images to be captured and digitalized were visually selected. In the two RV sections from each animal, different fields were captured, chosen where the highest number of cells could be visualized on cross section. For each ventricle analyzed, 50 cells were measured.

The cardiomyocytes selected were transversally sectioned. They were round with a central nucleus, located in the subendocardial layer of the RV muscle wall. This was to maximize the uniformity of the set of cardiomyocytes in the different groups. The mean sectional areas obtained for each group were used as an indicator of cell size.^29^

### Fractal dimension

To analyze the fractal dimension of the right ventricle, the photographed slides were binarized for reading, and the fractal dimension was estimated by using box-counting and the ImageJ image processing program (National Institutes of Health, USA), available free of charge at http://rsbweb.nih.gov/ij/.^11^

**Figure 1:**
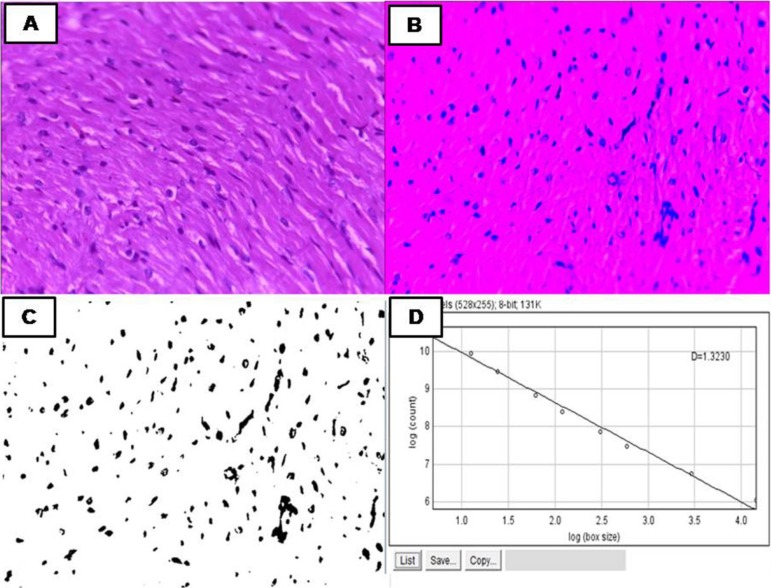
A) Histological section of the right ventricle stained with Hematoxylin and Eosin (HE), 40x magnification. B) Binarization process. C) HE image after binarization. Cell nuclei are stained black, while cytoplasm, cell membrane and other cell elements are white. D) Linear regression: sliding an r-sided square box over an image in an overlapping pattern, the box side r being progressively smaller; Nr is the number of r-sided boxes necessary to overlap the image, at each side size chosen. Fractal dimension is the inclination of the regression line for the two log values. Mann-Whitney test was used.

That program considers box-counting in two dimensions, allowing quantification of the distribution of pixels in the space, but does not consider image texture. Therefore, two images with the same distribution of pixels, one binarized and the other in shades of gray, will have the same fractal dimension. Thus, the analysis of the fractal histological slides is based on the relation between the resolution and the scale assessed, and the result can be quantitatively expressed as the fractal dimension of the object [FD = (Log Nr / log r-1), where Nr is the number of equal elements necessary to cover the original object, and r is the scale applied to the object (Figure 1).^30^ Therefore, the fractal dimension calculated with ImageJ program will always be between 0 and 2, not distinguishing different textures.

### Statistical analysis

Data were expressed as mean ± standard deviation and median (minimum - maximum). To analyze data normality, Shapiro Wilk test was used. The groups were compared by using non-paired Student *t* test (PAVm, RVW/FBW, LVW/FBW and cardiomyocyte area) or Mann-Whitney test (PAT, EJT, RAW/FBW and fractal analysis), depending on data normality. The null hypothesis was rejected at 0.05 significance level. The analyses were performed with the GraphPad Prism® statistical program, version 5.0.

## Results

### Echocardiographic functional assessment

Table 1 shows the RV echocardiographic parameters of the groups assessed. Group M animals showed increased pulmonary arterial pressure and RV functional worsening.

### Assessment of anatomical and histomorphometric parameters

Table 2 shows the anatomical and histomorphometric data of groups C and M. Group M had higher values of atrialW/FBW and RV/FBW indices than group C. A significant increase in the cross-sectional area of RV cardiomyocytes was also observed in group M animals (Table 2).

**Table 2:** Anatomical and histomorphometric data expressed as mean ± standard deviation, median, minimum and maximum values of the groups studied

VARIABLES	Group C	Group M	P value
AW/FBW (g)	0.20 ± 0.03 (0.2) [0.18 - 0.28]	0.35 ± 0.16 (0.31) [0.20 - 0.70]	0.0030
RVW/FBW (g)	0.44 ± 0.05 (0.43) [0.37 - 0.53]	0.81 ± 0.30 (0.78) [0.47 - 1.18]	0.0040
LVW/FBW (g)	1.85 ± 0.07 (1.85) [1.73 - 1.95]	1.87 ± 0.11 (1.86) [1.72 - 2.00]	0.7072
Area (µm^2^)	61.49 ± 7.47 (58.62) [54.91–75.11]	103.90 ± 20.82 (106.4) [78.20 – 129.50]	0.0001

C: control; M: monocrotaline; AW: atrial weight; FBW: final body weight; RVW: right ventricular weight; LVW: left ventricular weight; g: grams. (AW/FBW: Mann-Whitney test; RVW/FBW, LVW/FBW and Area - non-paired t test)

On postmortem examination, group M animals showed no signs of heart failure, such as ascitis, pleural effusion, and liver congestion.

### Fractal dimension

**Figure 2:**
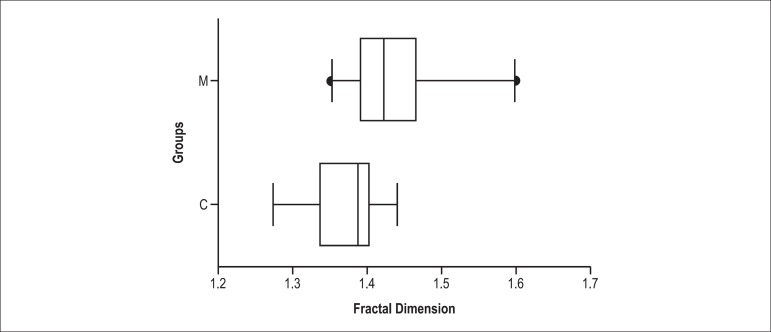
Fractal dimension analysis. C: control; M: monocrotaline; * p < 0.05. (Mann-Whitney test)

Group M had higher fractal dimension values than group C (1.43 ± 0.06 vs. 1.37 ± 0.045; p = 0.0012; Figure 2).

## Discussion

The results of the present study show that monocrotaline-induced PAH in rats caused an increase in RV fractal dimension, in addition to RV hypertrophy and contractile function worsening.

Experimental models of PAH are often used to assess and understand the pathophysiological mechanisms of that disease.^5,6,19,31^ The use of monocrotaline to induce PAH is a well-established model.^32^ Monocrotaline induces injury to the pulmonary capillaries, with an increase in pulmonary vascular resistance and ventricular afterload, causing progressive pathological RV remodeling with hypertrophy induction, increased interstitial fibrosis, cardiac dysfunction and heart failure.^33^

Group M animals had RV hypertrophy and developed cardiac dysfunction with RV systolic function worsening, suggesting the development of PAH and confirming the role of monocrotaline in triggering that disease. In addition, other studies using the same experimental model in rats have reported RV hypertrophy and myocardial contractile function worsening.^5,6,19,31^

The fractal dimension of the animals with PAH increased as compared to that of controls, suggesting that animals with RV hypertrophy and cardiac dysfunction have higher fractal dimension. To our knowledge, this is the first study assessing fractal dimension in RV dysfunction caused by monocrotaline-induced PAH in rats.

Fractal dimension is a useful method to characterize irregular structures.^34^ It counts the effective number of degrees of freedom in the dynamic system, quantifying, therefore, its complexity.^8,13^ One can infer that images evidencing higher fractal dimension are, thus, more complex.^8,10,13^

Histological changes caused by RV overload alter the amount and distribution of the information contained in the histological slide. The most commonly used histological methods to analyze cardiac remodeling assess either the structures qualitatively or depend on the proper visualization of particular aspects, such as cross-sectional cardiomyocyte location.^3,19,29^ In that context, fractal dimension would prevent that difficulty by adding a numerical value to the analysis, thus, allowing quantification of tissue structural changes. In addition, that method prevents possible errors of interobserver variations.^11^

Fractal dimension has been used in several medical areas, such as oncology, neurology, ophthalmology, radiology and cardiology,^9,10^ to characterize and identify irregular and complex structures.^11^ In addition, fractal concepts have been incorporated into models of biological processes, such as epithelial cell growth, detection of DNA encoding regions, blood vessel growth, periodontal disease and viral infections.^8,34^

The results of this study indicate that fractal dimension analysis can be used to characterize cardiac ventricular changes in such a prevalent and disabling disease as PAH is.

It is worth noting that, at an initial phase of cardiac remodeling without heart failure, which is the progression of PAH, fractal dimension analysis was sensitive to detect ventricular changes, showing its importance to the early identification of those changes.

One limitation of this study is that fractal dimension quantifies the degree of complexity of the image, and, thus, that technique compares to neither Western-Blot nor RT-qPCR, which can quantify the total level of proteins and myocardial gene expression, but neither its distribution nor degree of complexity in the tissue.

Further studies are necessary in human beings with PAH in the phase of ventricular dysfunction to validate and corroborate the results of this study, and, thus to widen the knowledge on that disease and on new clinical perspectives for its early treatment.

## Conclusion

The results confirm the PAH-induced cardiac dysfunction and point to the fractal dimension increase in cardiomyocytes of rats with monocrotaline-induced PAH, fractal dimension being an effective method to assess cardiac morphological changes induced by ventricular dysfunction.
